# Estimating the effects of variation in viremia on mosquito susceptibility, infectiousness, and *R_0_* of Zika in *Aedes aegypti*

**DOI:** 10.1371/journal.pntd.0006733

**Published:** 2018-08-22

**Authors:** Blanka Tesla, Leah R. Demakovsky, Hannah S. Packiam, Erin A. Mordecai, Américo D. Rodríguez, Matthew H. Bonds, Melinda A. Brindley, Courtney C. Murdock

**Affiliations:** 1 Department of Infectious Diseases, College of Veterinary Medicine, University of Georgia, Athens, Georgia, United States of America; 2 Center for Tropical and Emerging Global Diseases, University of Georgia, Athens, Georgia, United States of America; 3 Biology Department, Stanford University, Stanford, California, United States of America; 4 Centro Regional de Investigación en Salud Pública, El Instituto Nacional de Salud Pública, Tapachula, Chiapas, México; 5 Department of Global Health and Social Medicine, Harvard Medical School, Boston, Massachusetts, United States of America; 6 Department of Population Health, University of Georgia, Athens, Georgia, United States of America; 7 Center for Vaccines and Immunology, University of Georgia, Athens, Georgia, United States of America; 8 Odum School of Ecology, University of Georgia, Athens, Georgia, United States of America; 9 Center of Ecology of Infectious Diseases, University of Georgia, Athens, Georgia, United States of America; 10 River Basin Center, University of Georgia, Athens, Georgia, United States of America; University of Washington, UNITED STATES

## Abstract

Zika virus (ZIKV) is an arbovirus primarily transmitted by *Aedes* mosquitoes. Like most viral infections, ZIKV viremia varies over several orders of magnitude, with unknown consequences for transmission. To determine the effect of viral concentration on ZIKV transmission risk, we exposed field-derived *Ae*. *aegypti* mosquitoes to four doses (10^3^, 10^4^, 10^5^, 10^6^ PFU/mL) representative of potential variation in the field. We demonstrate that increasing ZIKV dose in the blood-meal significantly increases the probability of mosquitoes becoming infected, and consequently disseminating virus and becoming infectious. Additionally, we observed significant interactions between dose and days post-infection on dissemination and overall transmission efficiency, suggesting that variation in ZIKV dose affects the rates of midgut escape and salivary gland invasion. We did not find significant effects of dose on mosquito mortality. We also demonstrate that detecting virus using RT-qPCR approaches rather than plaque assays potentially over-estimates key transmission parameters, including the time at which mosquitoes become infectious and viral burden. Finally, using these data to parameterize an *R*_*0*_ model, we showed that increasing viremia from 10^4^ to 10^6^ PFU/mL increased relative *R*_*0*_ 3.8-fold, demonstrating that variation in viremia substantially affects transmission risk.

## Author summary

The number of people at risk for contracting Zika virus (ZIKV) is difficult to estimate accurately because most infected hosts are asymptomatic and the relationship between variation in host viremia and transmission to local mosquitoes is unclear. Controlling ZIKV transmission remains a major challenge due to lack of basic information on transmission mechanisms and gaps in mechanistic models. Therefore, our study highlights the importance of variation in viral concentration that current modeling efforts ignore, which will enhance our ability to predict the number of people at risk for arbovirus infection, overall disease transmission, and the efficacy of current and future intervention strategies. We demonstrated that increased concentration of ZIKV in the blood significantly increases the probability and the rate at which mosquitoes become infectious, which increases the risk of ZIKV transmission.

## Introduction

Although discovered in 1947 [1], Zika virus (ZIKV) has recently become a public health concern due to its rapid spread and newly identified teratogenic effects [2]. Shortly after isolation from a rhesus macaque in Uganda, the virus caused several mild infections in humans [3, 4]. ZIKV infections remained inapparent until the first major outbreak in 2007 on the island of Yap [5]. The virus further spread across the Pacific, where it was first associated with Guillain-Barré syndrome during the 2014 French Polynesian outbreak [6]. In 2015, transmission was confirmed in Brazil [7], after which the virus spread rapidly across the Americas [8]. ZIKV was declared a “public health emergency of international concern” by WHO in 2016 due to rapid spread and increases in complications associated with congenital Zika virus syndrome [9].

The primary route of ZIKV transmission is through the bite of *Aedes* mosquitoes, but the virus can also be spread vertically [2], sexually [10], and through blood transfusion [11]. The principal urban vector in the Americas is *Ae*. *aegypti*, while *Ae*. *albopictus* is believed to be a secondary vector [12]. Although most cases of ZIKV infection are asymptomatic [5], 20% of individuals develop symptoms associated with Zika fever [13]. Currently, human viremia is not well characterized. Studies suggest that ZIKV viremia in the blood is lower than other arboviruses and does not significantly differ between symptomatic and asymptomatic patients [14]. In arboviral systems such as dengue, variation in viremia across infectious human hosts influences the number of mosquitoes that become infectious [15], yet this has only been minimally explored in the ZIKV system [16–18]. Further, the impact of variation in host viremia on overall transmission has yet to be adequately addressed.

The number of people at risk for contracting ZIKV or other similarly transmitted arboviruses (e.g., dengue and chikungunya) is difficult to estimate accurately because most ZIKV infected hosts are asymptomatic, the distribution of hosts with varying viremia is unknown, and the relationship between variation in host viremia and transmission to local mosquito populations is unclear. *R*_*0*_ (the basic reproductive number of a pathogen) represents the expected number of secondary cases that result from a single infection in a susceptible population and is comprised of a combination of human, mosquito, and pathogen traits [19, 20]. *R*_*0*_ models allow for the estimation of the epidemic spread of pathogens [19–21], are commonly used to assess the effectiveness of mosquito control strategies [22–25], and are routinely used to predict the coverage required for successful vaccination programs [26–28]. Yet, our current ability to estimate the number of human hosts at risk or to control ZIKV transmission is limited by the lack of basic information on transmission mechanisms, leading to gaps in mechanistic models, the most fundamental of which is *R*_*0*_. To address this limitation, we conducted experiments to assess the effect of variation in viral dose on vector competence, the extrinsic incubation rate (EIR), and mosquito survival. We used these results to parameterize a mechanistic *R*_*0*_ model and to estimate the number of infectious bites contributed by mosquito populations feeding on hosts with varying viremia.

## Methods

### Mosquito rearing

We generated an outbred field-derived population of *Ae*. *aegypti* mosquitoes from ovitrap collections in Tapachula, Chiapas, Mexico, 2016. Larvae were reared in trays (200 larvae/1L ddH_2_O) and fed with 4 fish food pellets (Hikari Cichlid Cod Fish pellets). Larvae and adults were kept under standard insectary conditions at 27°C ± 0.5°C, 80% ± 10% relative humidity, and a 12:12 hours light:dark diurnal cycle. Mosquitoes were maintained on human blood (Interstate Blood Bank) and provided with 10% sucrose *ad libitum*. F2—F4 generations of mosquitoes were used for all downstream experiments.

### Virus culture

For all mosquito infections, we used the ZIKV MEX1-44 strain obtained from the University of Texas Medical Branch Arbovirus Reference Collection. The virus was isolated from *Ae*. *aegypti* in 2016 from Chiapas, Mexico and passaged in Vero cells nine times. Vero cells were maintained in Dulbecco’s modified Eagle’s medium (DMEM) supplemented with 5% fetal bovine serum (FBS) at 37°C and 5% CO_2_. The virus was harvested four days after inoculation and stored at -80°C for at least seven days before titrating. Titers were determined by standard plaque assays on Vero cells as previously described [29], and expressed in plaque-forming units per milliliter (PFU/mL). Virus tested negative for *Mycoplasma* contamination using MycoSensor PCR Assay Kit (Agilent).

### Experimental mosquito infections

All ZIKV infections were performed under ACL3 conditions at the University of Georgia, Athens, GA, USA. Two days prior to the infectious feed, we separated 1 to 3-day-old female mosquitoes and sorted them into eight 64 oz paper cups with 200 mosquitoes per cup. After separation, females were provided water and transported to the ACL3 facility. On the day of infection, we prepared infectious and control blood-meals by washing human blood three times in RPMI medium. We then mixed 50% red blood cells with 33% DMEM, 20% FBS, 1% (wt/vol) sucrose, and ATP to a final concentration of 5 mmol/L. The blood-mixture was then mixed with virus at a 1:1 ratio. We fed three to five day-old mosquitoes on a water-jacketed membrane feeder containing uninfected (n = 80) or infectious blood-meals (n = 400 per treatment) with a final concentration of 10^3^ (M = 4*10^3^, SD = 2.12*10^3^), 10^4^ (M = 2.9*10^4^, SD = 2.65*10^3^), 10^5^ (M = 2.27*10^5^, SD = 1.34*10^5^) or 10^6^ (M = 2.13*10^6^, SD = 1.45*10^6^) PFU/mL for 30 min. We then randomly distributed 200 engorged mosquitoes from each dose treatment into 16 oz paper cups (n = 40 per cup) for destructive sampling every 4 days post-infection (dpi). An additional 40 mosquitoes engorged with uninfected blood were placed in a 16 oz paper cup to track mortality. At each sampling time point, 20 mosquitoes from each dose treatment group were removed from one cup for forced salivations (n = 100 total per dose treatment group, n = 400 per experiment). Every two days, we recorded the number of dead and alive mosquitoes across all the cups. The mosquitoes were housed at 27°C ± 0.5°C, 70% ± 5 relative humidity, and a 12:12 hours light:dark photoperiod with *ad libitum* access to 10% sucrose solution and water for up to 20 days. Three full biological replicates of this experiment were performed (n = 1200 total; S1 Fig).

### Quantifying mosquito infection via forced salivations

To determine the proportion of mosquitoes that were infected with ZIKV, had disseminated infections, and were infectious, we processed 20 mosquitoes per treatment group on days 4, 8, 12, 16 and 20 post-infection. Mosquitoes were cold anesthetized and kept on ice until their legs and wings were removed. After immobilization, we transferred the mosquitoes to a hot plate (35°C) and placed the proboscis of each mosquito into a pipet tip containing 35 μL FBS with 3 mmol/L ATP and red food dye, after which they were allowed to salivate for 45 min. After salivation, mosquitoes were decapitated, and bodies, heads, and saliva were individually placed into tubes containing 600 μL of DMEM with 1x antibiotic/antimycotic. Bodies and heads were homogenized in a QIAGEN TissueLyzer at 30 cycles/second for 30 seconds, and centrifuged at 17,000xg for 5 minutes at 4°C. Samples were then assessed for the presence/absence of virus with plaque assays.

### RT-qPCR analysis

To compare plaque assays with quantitative reverse transcription PCR (RT-qPCR), we performed RT-qPCR on saliva samples from mosquitoes exposed to 10^5^ and 10^6^ PFU/mL at days 4 and 20 post-infection. Viral RNA was extracted from saliva samples (QIAamp Viral RNA Mini Kit, Qiagen) and reverse-transcribed to cDNA (High Capacity RNA-to-cDNA Kit, Applied Biosystems). ZIKV genome copies were measured with RT-qPCR reaction assay using TaqMan Gene Expression Master Mix (Applied Biosystems), primers (F: ZIKV 1086, R: ZIKV 1162c; Invitrogen Custom Primers) and probes (ZIKV 1107-FAM; TaqMan MGB Probe) [30]. Each sample was analyzed in duplicate, and each assay contained a standard curve (ZIKV molecular clone), no template, and no primer controls. We extrapolated ZIKV copy numbers from the generated standard curve using the Applied Biosystems protocol. The limit of detection was experimentally established to be 30 copies (10^−16^ g). Final copy numbers were adjusted by back-calculations to the total RNA and cDNA volume and expressed as copies per saliva sample.

### Statistical analysis

From these data, we ran two general sets of analyses. The first set of analyses explored the effects of dose, day post-infection (dpi), and their interaction on the numbers of mosquitoes infected, with disseminated infections, and infectious out of the total number of mosquitoes exposed. These analyses were performed to estimate the effects of variation in viral dose on vector competence and the extrinsic incubation period (the rate of becoming infectious), both crucial parameters in estimating dose effects on transmission potential (*R*_*0*_) and the force of infection. The second set of analyses investigated the effects of dose, dpi, and their interaction on the numbers of mosquitoes with disseminated infection and that are infectious out of total number of mosquitoes successfully infected, as well as on overall viral burden. These analyses are important for exploring the effects of variation in viral dose on different aspects of the virus infection in the mosquito (e.g. virus escape from the midgut and salivary gland tissue barriers) and for inferring how dose affects the mosquito-virus interaction.

We used mixed effects generalized linear models (IBM SPSS Statistics 1.0.0.407) to estimate the effects of ZIKV dose, dpi, and their interaction (fixed factors) on the number of mosquitoes, out of total mosquitoes exposed, that are infected (positive bodies: negative binomial distribution, log link function), have disseminated infection (positive heads: normal distribution, identity link function), and are infectious (positive saliva: normal distribution, identity link function), as well as viral burdens in the saliva (normal distribution, log link function). Similar models were also constructed to assess dose and dpi effects on dissemination efficiency (of those infected, the number of mosquitoes with disseminated infections; negative binomial, log link function) and transmission efficiency (of those infected, the number of mosquitoes with positive saliva: negative binomial distribution, log link function); however, we weighted these models by the number of mosquitoes with positive bodies as this varied due to ZIKV dose effects on midgut infection. Finally, we used a Cox mixed effects model (R version 3.3.3, package ‘coxme’ [31]) to estimate the effects of ZIKV infection (exposed / unexposed), dose, and their interaction on the daily probability of mosquito survival. Experimental replicate was included in all models as a random effect. Model fit and distributions were determined based on Akaike Information Criterion (AIC), the dispersion parameter, and by plotting residuals. Sequential Bonferroni tests were used to assess the significance of pairwise comparisons when relevant, and *p*-values greater than 0.05 were considered non-significant.

### Mechanistic *R*_*0*_ model

To estimate the effects of dose on transmission risk, we used two different approaches. First, we calculated relative *R*_*0*_ as a function of dose (*x*) since the absolute magnitude of *R*_*0*_ depends on other factors not considered here. We modified a function of *R*_*0*_ used in previous work [20] (Eq 1):

$$R_{0}(x)=\sqrt{\frac{a^{2}\;bc(x)\;exp(-\mu (x)/EIR(x))\;EFD\;p_{EA}\;MDR}{N\;r\;{\mu (x)}^{3}}}$$ (1)

with parameters for the daily biting rate (*a*), vector competence (*bc*), the daily adult mosquito mortality rate (*μ*), the extrinsic incubation rate (*EIR*), the eggs per female per day (*EFD*), the probability of egg to adult survival (*p*_*EA*_), the mosquito development rate (*MDR*), the density of humans (*N*), and the human recovery rate (*r*). For parameters we did not directly estimate (*a*, *EFD*, *p*_*EA*_, *MDR*), we used estimates generated by Mordecai et al. [20] for *Ae*. *aegypti* at 27°C and assumed the human recovery rate to be the inverse of the average number of days ZIKV is detectable in the blood (6 days [32]). We used the experimental infection data to estimate dose-dependent vector competence (*bc*), *EIR*, and daily mortality rate (*μ*) as follows. We fit logistic growth models to the proportion of infectious mosquitoes (*Y*) versus dpi (*t*) for each viral dose (*x*) (Eq 2),

$$Y(t)=\frac{bc(x)}{1+e^{-k(t-EIP(x))}}$$ (2)

using the “nls” package in R [33]. Vector competence was defined as the maximum proportion of infectious mosquitoes out of total exposed per dose (the asymptote, *bc*), *EIR* was estimated as the inverse of the extrinsic incubation period (*EIP*, the inflection point or the time it takes the mosquito population to achieve 50% of maximum vector competence), and *k* reflects the instantaneous rate of increase (slope at the inflection point). Then, to estimate the daily probability of mosquito mortality (*μ*) we fit a variety of non-linear curves (exponential, log-linear, Weibull, and Gompertz) to the daily survival probabilities of mosquitoes exposed to different doses with the “flexsurv” package in R [34]. We used AIC to determine the best performing model and calculated the area under the curve to estimate the average lifespan (*lf*) of mosquitoes exposed to varying doses. The average daily probability of mortality was then estimated as the inverse of the dose-specific lifespan (1/*lf*).

Second, we performed an alternative calculation of transmission risk following previously described methods [35] to account for the substantial variation in infection outcomes observed across mosquitoes exposed to a given dose. Briefly, we multiplied the best fitting non-linear functions describing the daily relationship between survival and the proportion of infectious mosquitoes for each dose treatment, resulting in the number of infectious days/dose. We then estimated the area under the curve of the resulting function and multiplied by the daily biting rate (*a*) [20] to calculate the number of infectious mosquito bites generated for each dose treatment for a mosquito population of a given size (n = 100).

## Results

### The effect of viral dose on vector competence and EIR

**Fig 1:**
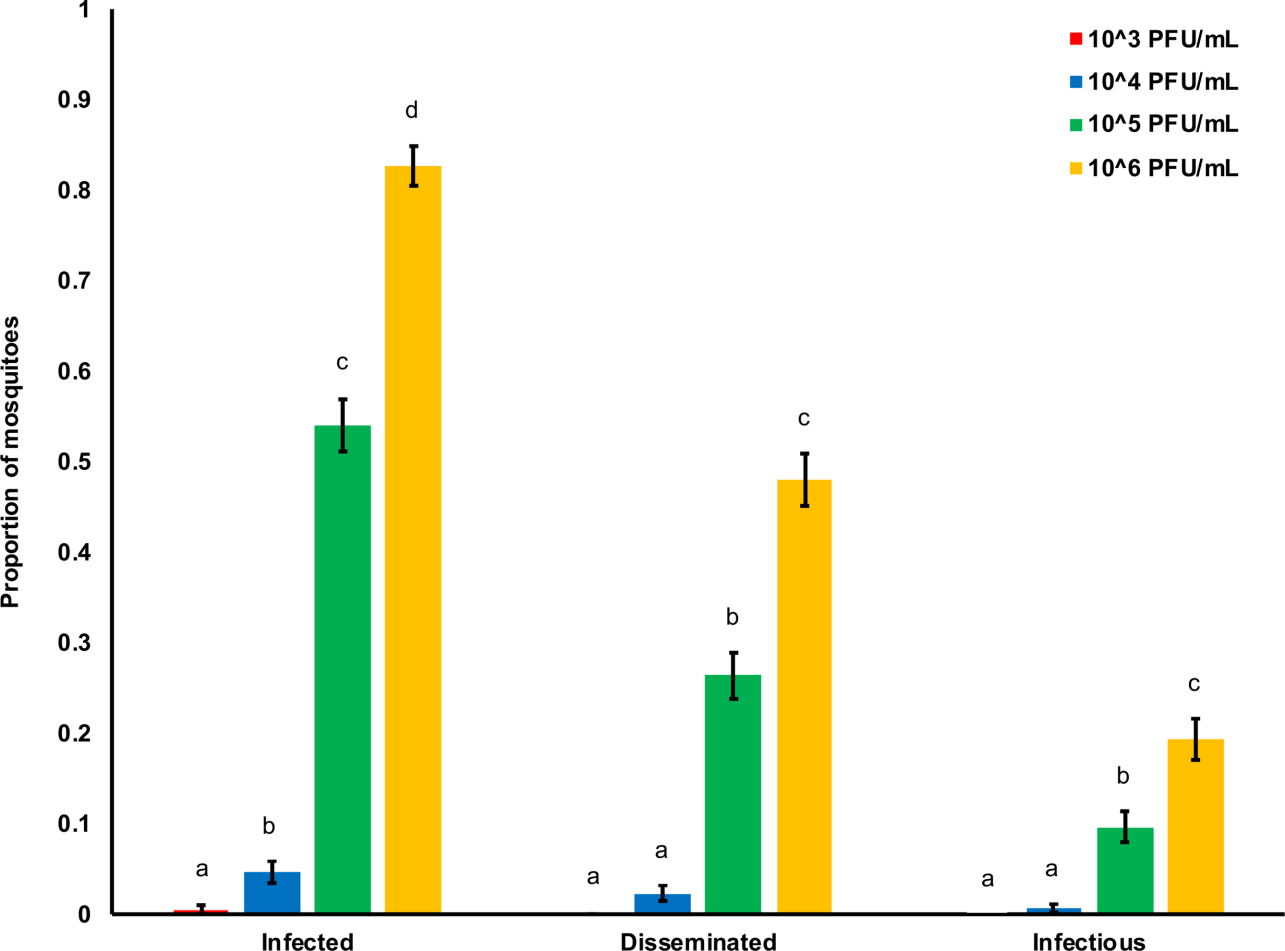
ZIKV dose and the proportion of mosquitoes infected, with disseminated infections, and infectious. Relationship between the ZIKV dose (10^3^, 10^4^, 10^5^, and 10^6^ PFU/mL) and the proportion of mosquitoes infected (ZIKV positive bodies), with disseminated infections (ZIKV positive heads), and infectious (ZIKV positive saliva) out of the total number of exposed mosquitoes. For each category, results with no common letters were significantly different (*p* ≤ 0.05) and whiskers on each bar represent the standard error of the mean.

**Fig 2:**
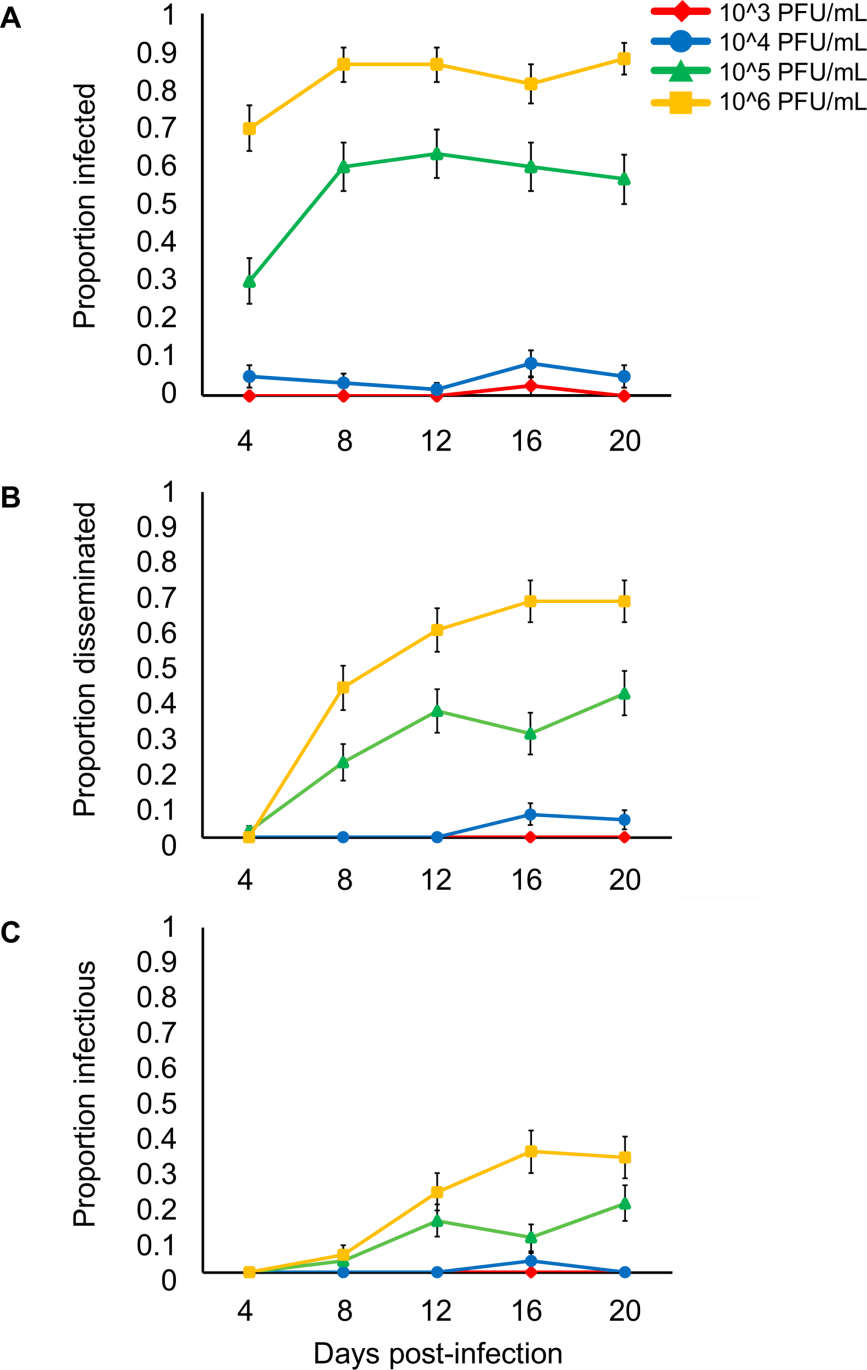
Days post-infection and the proportion of mosquitoes infected, with disseminated infections, and infectious. The relationship between days post-infection (4, 8, 12, 16, 20) and the proportion of mosquitoes infected (A), with disseminated infections (B), and infectious (C) after exposure to four different viral doses (10^3^, 10^4^, 10^5^, and 10^6^ PFU/mL). Whiskers on each bar represent the standard error of the mean.

To investigate how variation in ZIKV dose affects vector competence, transmission efficiency, and the extrinsic incubation period in *Ae*. *aegypti*, we orally infected mosquitoes with four different viral concentrations (10^3^, 10^4^, 10^5^ and 10^6^ PFU/mL) reflecting viremia in ZIKV-infected humans. We found that the mean proportion of infected mosquitoes, mosquitoes with disseminated infections, and infectious mosquitoes significantly increased with increasing viral dose (Table 1, Fig 1). The infectious dose required to infect 50% of the mosquito population (ID_50_) was 10^4.98^ PFU/mL. We also observed a significant effect of days after infection on the probability that mosquitoes had disseminated infection or became infectious, but not on the probability that they became infected (Table 1). At the highest doses (10^5^ and 10^6^ PFU/mL), the virus was detectable in mosquito bodies at all tested time points (Fig 2A). On average, more than 4 days were required for ZIKV to disseminate into the head (Fig 2B) and more than 8 days to be present in the saliva (Fig 2C). Finally, the significant interaction between ZIKV dose and days post-infection indicates that increases in viral concentration significantly increased the rate at which mosquitoes disseminate infection and become infectious (Table 1, Fig 2). These results suggest that mosquitoes feeding on human hosts with varying levels of circulating virus could experience both different probabilities of infection and overall infection dynamics.

**Table 1:** The effect of dose, day, and the potential interaction on mosquito infection, dissemination, infectiousness, and dissemination and transmission efficiencies.

response variables	dose			day			dose x day		
	F	d.f.	*p*-value	F	d.f.	*p*-value	F	d.f.	*p*-value
probability of infection	33.898	3	**<0.0001**	0.004	4	1	0.501	12	0.9
probability of dissemination	52.61	3	**<0.0001**	11.929	4	**<0.0001**	4.295	12	**<0.0001**
probability of infectiousness	22.86	3	**<0.0001**	7.82	4	**<0.0001**	3.45	12	**0.002**
dissemination efficiency	0.852	3	0.466	1.102	4	0.355	2.328	8	**0.019**
transmission efficiency	0.011	3	0.998	0.027	4	0.999	3.862	8	**<0.0001**

Results from generalized linear mixed effects models examining the effects of dose, day, and the interaction on the numbers of mosquitoes infected (ZIKV positive bodies out of total number exposed), with disseminated infections (ZIKV positive heads out of total number exposed), infectiousness (ZIKV positive saliva out of total number exposed), and measures of dissemination (ZIKV positive heads out of positive bodies) and transmission (ZIKV positive saliva out of positive bodies) efficiency.

### The effect of viral dose on ZIKV transmission efficiency

**Fig 3:**
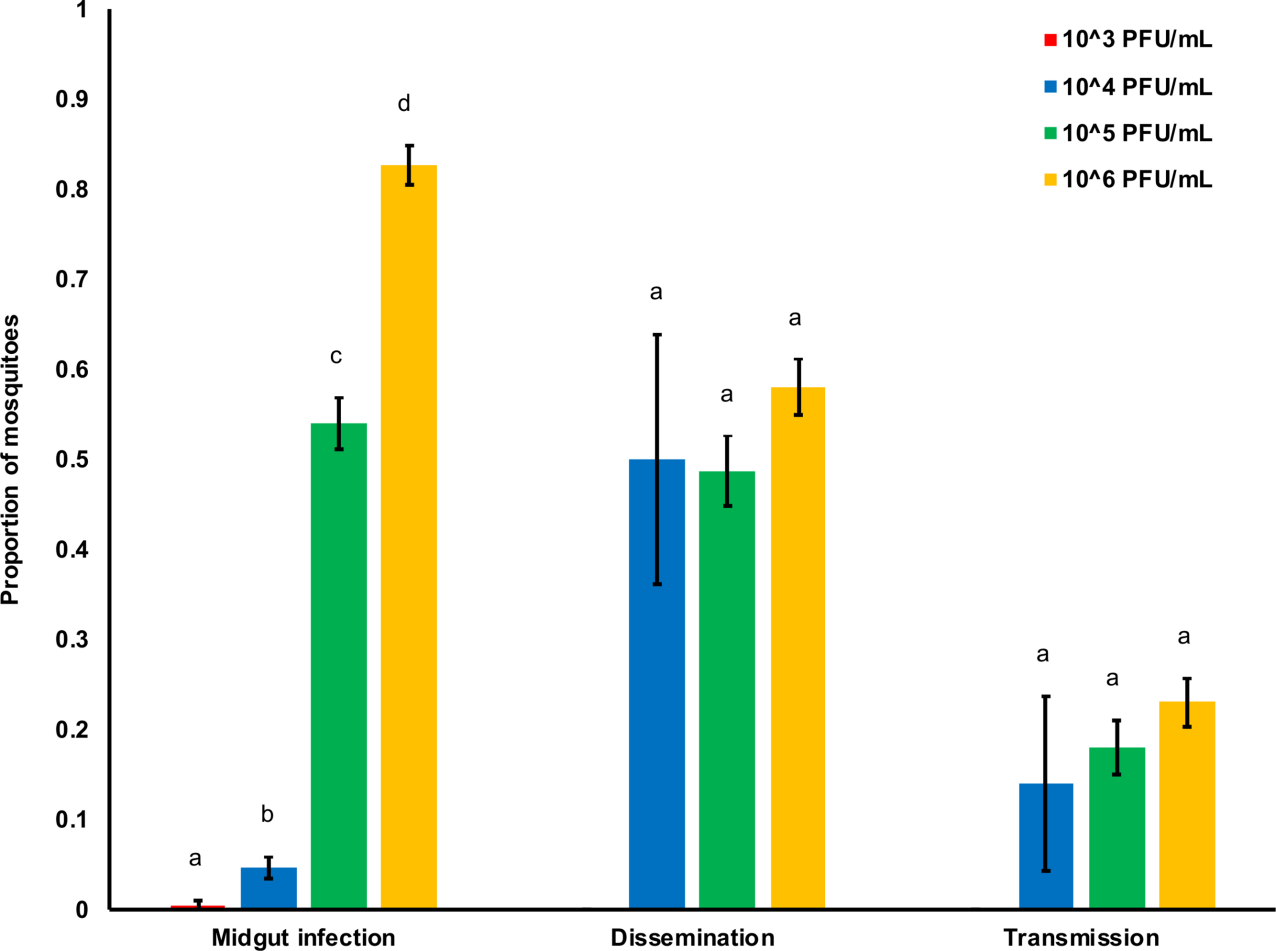
ZIKV dose and the efficiency of midgut infection, dissemination, and transmission. Relationship between the ZIKV dose (10^3^, 10^4^, 10^5^, and 10^6^ PFU/mL) and the efficiency of midgut infection (proportion of ZIKV positive bodies out of total number exposed), dissemination (proportion of ZIKV positive heads out of all positive bodies), and transmission (proportion of ZIKV positive saliva out of all positive bodies). For each category, results with no common letters were significantly different (*p* ≤ 0.05) and whiskers on each bar represent the standard error of the mean.

We measured the effect of viral dose on transmission efficiency; specifically, the proportion of infected mosquitoes that have disseminated infection (dissemination efficiency) and that became infectious (transmission efficiency). Despite the significant effects of dose and days post-infection on the number of mosquitoes that disseminate infection and that are infectious out of the total number of mosquitoes exposed, these main effects did not affect measures of dissemination and transmission efficiency (Fig 3, Table 1). However, we did identify a significant interaction between ZIKV dose and days post-infection for both response variables (Table 1, S2 Fig). This interaction demonstrates that variation in viral dose significantly affects the rate at which virus escapes the midgut and salivary gland barriers, with increases in viral dose resulting in more rapid dissemination and overall infectiousness.

### The effect of ZIKV infection on mosquito survival

To determine if ZIKV infection and viral dose altered the probability of survival in *Ae*. *aegypti* mosquitoes, we included uninfected blood-fed controls in the study. We did not find any significant differences in the probability of survival between uninfected and ZIKV infected mosquitoes. Further, we observed no effects of increasing viral dose on mosquito survival among the infected mosquitoes (Table 2). On average, *Ae*. *aegypti* fed on viral doses of 10^3^, 10^4^, 10^5^, and 10^6^ PFU/mL experienced an average lifespan (*lf*) of 27, 24, 30, and 29 days, respectively.

**Table 2:** The effects of ZIKV dose on the daily probability of mosquito survival.

dose	z	*p*-value
uninfected	-1.44	0.15
10^3^	0.64	0.52
10^4^	1.05	0.29
10^5^	-0.03	0.97
10^6^	-0.55	0.58

Results from Cox mixed-effects model examining the effects of ZIKV dose (10^3^, 10^4^, 10^5^, and 10^6^ PFU/mL) on the daily probability of mosquito survival.

### Comparison between plaque assays and RT-qPCR

**Fig 4:**
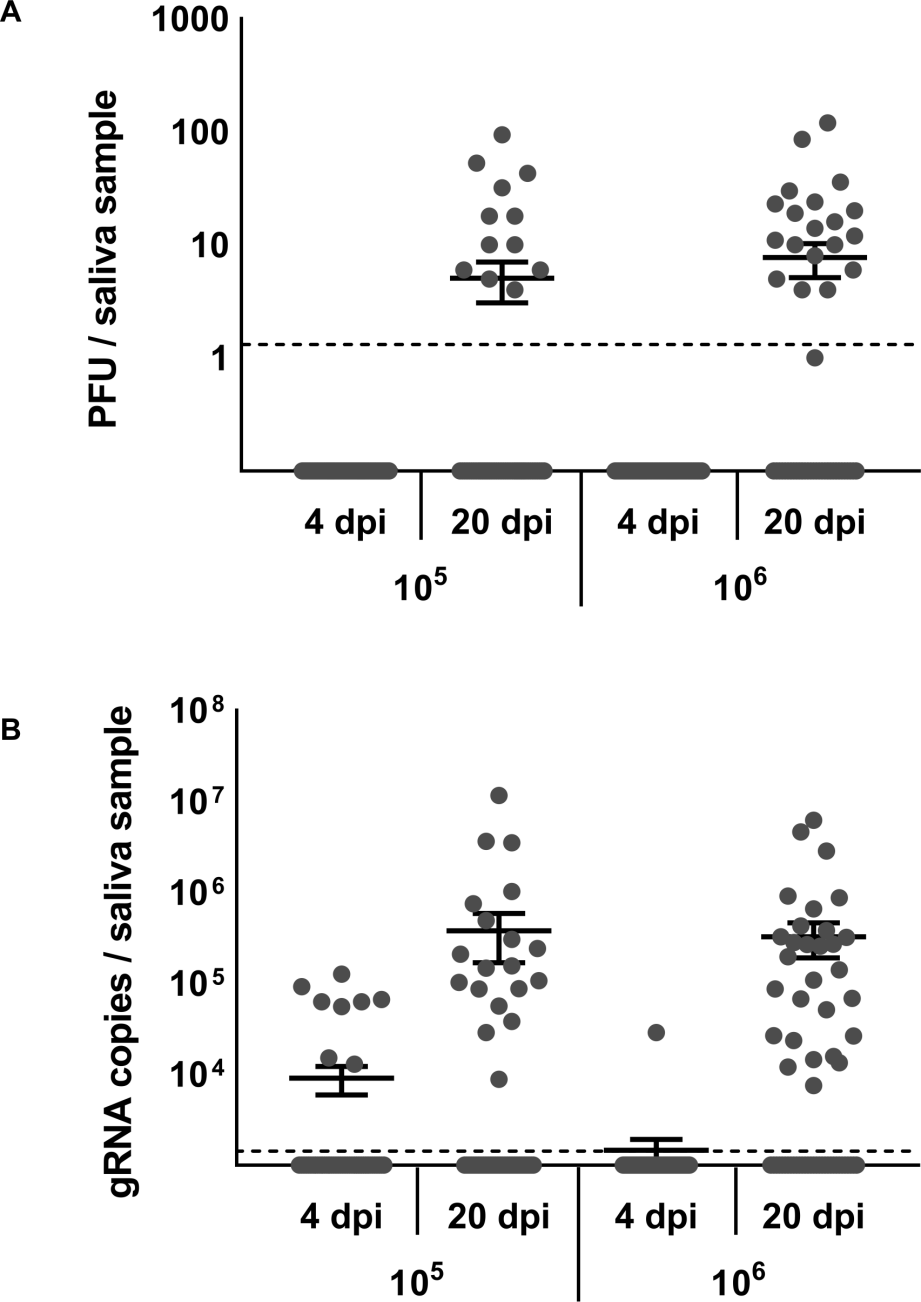
Viral loads in saliva determined by plaque assays and RT-qPCR. Viral load of ZIKV in saliva at 4 and 20 days post-infection (dpi) with 10^5^ and 10^6^ PFU/mL determined by standard plaque assays on Vero cells (A) and ZIKV-specific RT-qPCR (B). The limit of detection was experimentally established to be 3 plaque-forming units (PFU) for plaque assays and 30 gRNA copies for RT-qPCR.

Most studies use qPCR to assess mosquito infection status. This method not only detects infectious particles, but also detects the viral genomic RNA in the infected cells, producing high RNA values that do not reflect the levels of infectious particles in the sample. When comparing the performance of plaque assays and RT-qPCR to assess infection status, we included the two highest doses (10^5^ and 10^6^ PFU/mL) because we had few to no positive saliva samples from the 10^3^ and 10^4^ treatment groups. Overall, the two methods gave similar numbers of positive samples (Table 3); however, we can detect the presence of ZIKV genome in mosquito saliva using RT-qPCR methods as early as 4 dpi, which was never the case with plaque assays. In fact, infectious particles were rarely detected at 8 dpi with plaque assays. The number of infectious particles ranged from 3 (the limit of detection) to 120 PFU per sample and RNA molecules ranged from 10^4^ to 10^7^ gRNA copies (Fig 4A and 4B). Both detection methods show that viral concentration does not have a significant effect on ZIKV levels in the saliva. However, we do see a significant effect of days after infection on viral gRNA copies detected by RT-qPCR (Table 4).

**Table 3:** Numbers of positive saliva samples determined by RT-qPCR and plaque assays.

replicate	dose (PFU/mL)*	day	gRNA	PFU
1	10^5^	4	3	0
		20	7	4
	10^6^	4	1	0
		20	12	9
2	10^5^	4	2	0
		20	5	5
	10^6^	4	0	0
		20	10	7
3	10^5^	4	3	0
		20	7	3
	10^6^	4	0	0
		20	7	4

*Plaque-forming units per milliliter.

Numbers of positive saliva samples determined by RT-qPCR (gRNA) and plaque assays (plaque forming units, PFU) for 10^5^ and 10^6^ viral doses on days 4 and 20 post-infection for each experimental replicate.

**Table 4:** The effects of dose and day on the number of ZIKV gRNA copies and plaque-forming units.

factors	gRNA copies			plaque-forming units		
	F	d.f.	*p*-value	F	d.f.	*p*-value
dose	0.873	1	0.354	0.136	1	0.715
day	5.688	1	**0.021**	-	-	-

Results from generalized linear mixed effects models examining the effects of dose and day on the number of ZIKV gRNA copies vs. plaque-forming units

### The effect of viral dose on overall transmission

**Fig 5:**
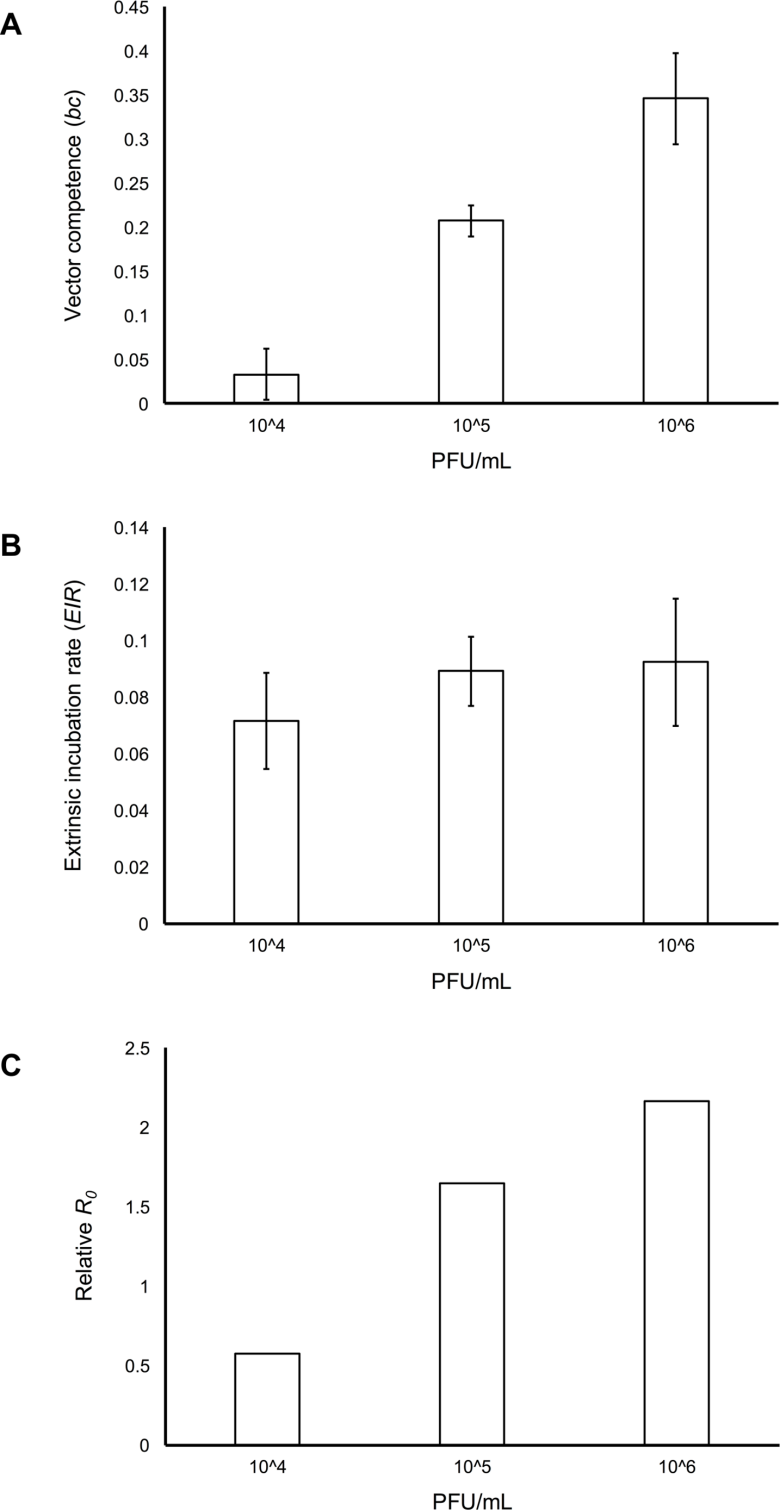
Viral dose and estimated vector competence, extrinsic incubation rate, and relative basic reproductive number *R*_*0*_. Relationship between viral dose (10^4^, 10^5^, and 10^6^ PFU/mL) and estimated vector competence (A), the extrinsic incubation rate (B), and relative basic reproductive number *R*_*0*_ (C).

**Fig 6:**
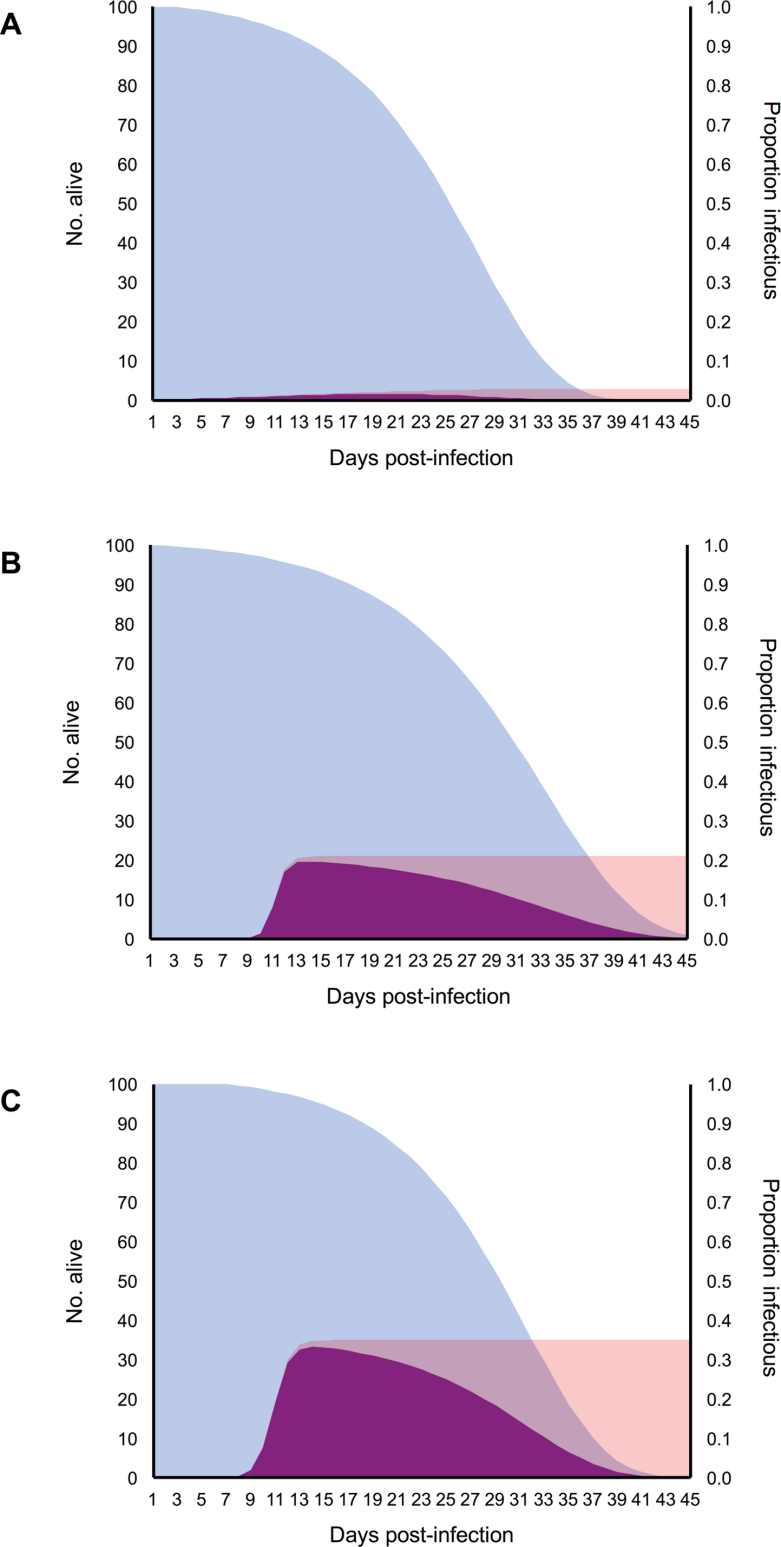
Daily proportion of mosquitoes alive, infectious, and both alive and infectious for mosquitoes exposed to different doses. Relationship between the daily proportion of mosquitoes alive (blue distributions), infectious (pink distributions), and those that are both alive and infectious (purple distributions) for mosquito populations exposed to 10^4^ (A), 10^5^ (B) and 10^6^ PFU/mL (C).

The maximum proportion of the mosquito population that became infectious (vector competence; *bc*) increased with viral dose (Fig 5A). In contrast, the estimated *EIR* did not differ substantially among mosquitoes fed different viral doses (Fig 5B), further suggesting that variation in infection dynamics with viral dose is driven primarily by positive dose effects on viral infection and escape from the midgut. This in turn resulted in increases in the relative transmission risk (*R*_*0*_) of mosquito populations feeding on hosts of increasing viremias (Fig 5C) and the relative number of infectious bites a human population would experience from a mosquito population of a given size (Fig 6).

## Discussion

Mosquito vectors are often exposed to hosts that individually vary in pathogen loads, which can result in variation in the proportion of the mosquito population that becomes infectious [15, 36]. To date, only a few studies have explored how viral concentration impacts measures of vector competence for ZIKV [16–18], and no studies have explicitly linked this source of variation to metrics of transmission risk. In this study, we demonstrate that *Ae*. *aegypti* populations exposed to increasing ZIKV concentrations exhibit increases in vector competence and EIR, which in turn results in substantial increases in relative transmission risk, measured as either *R*_*0*_ or the force of infection.

Consistent with previous studies [16–18], we show that increasing the blood-meal concentration of ZIKV increases the probability mosquitoes will become infected, which in turn increases the probability of mosquitoes disseminating infection and to become infectious. Vector competence of a mosquito is strongly affected by the ability of a particular arbovirus to infect and escape the midgut and salivary gland barriers [37]. As in other studies, we demonstrate that increases in viral concentration facilitates ZIKV infection and midgut barrier escape [38]. A dose of at least 10^4^ PFU/mL was required for dissemination, and higher concentrations resulted in a higher proportion of mosquitoes with disseminated infections at earlier time points. Further, we show that increases in viral concentration increase the EIR of ZIKV, consistent with other studies [18, 39], in part due to the positive effects of dose on the rate at which virus escapes the midgut and salivary gland tissue barriers. Finally, due to the lack of a main effect of dose on dissemination and overall transmission efficiency, we show that the effects of variation in viral dose on vector competence is largely driven by a carry-over effect of dose on initial midgut infection. This is not surprising considering the probability of dissemination and becoming infectious is first dependent on successful midgut infection, as well as subsequent midgut escape and salivary gland invasion, respectively.

Compared to previous studies of ZIKV infection in *Ae*. *aegypti*, we found higher infection rates and a lower infectious dose 50 (ID_50_). Our estimated ID_50_ (10^4.98^ PFU/mL) is much lower than previously reported ID_50_ 10^7.4^ PFU/mL [18]. There is a substantial evidence for ZIKV, dengue, and chikungunya that vector competence can vary across mosquito populations due to genotype-by-genotype (G x G) interactions [17, 18, 39–41]. Our higher infection rates could be due to the fact that we paired a Mexican ZIKV isolate with an *Ae*. *aegypti* population collected from the same region. Considering most ZIKV infected patients exhibit low viremia relative to other arboviruses, the mosquito-virus pairing may also explain why our infectious dose is more consistent with real-world viremias than previous estimates [17, 18, 42].

Mosquito longevity, along with EIR, are the strongest drivers of *R*_*0*_. Together these two parameters determine the duration of time a mosquito is alive and infectious. We found no effect of ZIKV infection or viral concentration on mosquito survival. Although mosquito mortality was not checked daily, and the ability to detect small effects is limited, it is generally assumed that mosquitoes are fairly tolerant of viral pathogens, allowing the virus to persist in the host without incurring fitness costs [43]. However, most studies, including ours, have been performed in laboratory settings under relatively optimal conditions. Thus, if the costs of infection on mosquito survival and reproduction reflect underlying physiological trade-offs, fitness effects may only manifest in studies that incorporate relevant environmental stressors (e.g. variation in environmental temperature, food availability, competition, etc.) [44, 45]. Finally, we assume that biting rates are equivalent between ZIKV-exposed and unexposed mosquitoes and with increasing ZIKV dose. However, there is evidence that exposure to malaria and dengue can alter mosquito feeding behavior and biting rates hosts experience [46]. This is potentially an important avenue for future research, especially if ZIKV infection reduces or increases mosquito biting rates at specific points during the infection process and if dose modifies these relationships.

To understand how variation in viral dose affects potential transmission risk, we used our infection and mortality data to parameterize a relative *R*_*0*_ model and determine the force of infection. Both *R*_*0*_ and the force of infection are important measures of disease spread, representing the number of secondary cases in a susceptible population and the rate at which susceptible individuals acquire infectious disease, respectively. In our study, we show that mosquito populations feeding on increasing viral doses contribute more infectious bites and produce more secondary ZIKV cases due to increased vector competence and the rate at which virus escaped the midgut. For example, increasing viremia from 10^4^ to 10^6^ PFU/mL increased relative *R*_*0*_ 3.8-fold and the number of infectious bites 18-fold. Although populations of mosquitoes in the field are exposed to multiple doses, this was an important first step for understanding the implications of dose-dependent transmission. Knowing transmission risk will vary with heterogeneity in host viremia, future studies should focus on characterizing the distribution of viremia in the host population and incorporating individual variation in infectiousness into mechanistic models of disease spread. Model predictions from some pathogen systems (e.g. SARS, measles, and smallpox) that account for individual variation in infectiousness differ greatly from predictions generated by average-based approaches [47].

We used plaque assays to determine infection status instead of RT-qPCR, a common technique used in other studies due to its rapidity and sensitivity [48]. However, because this method will detect all viral RNA in infected tissues, it can overestimate the actual number of infectious particles present. While we found the results of RT-qPCR and plaque assays to be highly correlated, the number of genomes detected by RT-qPCR was much higher than the number of plaque-forming units. We detected ZIKV genome in mosquito saliva (4 dpi) well before our first ZIKV infectious saliva sample was detected by plaque assay (8 dpi). Other studies using RT-qPCR methods have reported ZIKV in mosquito saliva as early as 3 dpi [16, 49]. Since virus can be transmitted only in the form of infectious particles, the use of RT-qPCR to determine transmission relevant phenotypes could lead to overestimates of transmission risk.

In general, ZIKV viremia does not differ between symptomatic and asymptomatic patients [14] and is on average lower than seen with other arbovirus systems [39, 50]. Contrary to our study, in the dengue and malaria systems, asymptomatic and pre-symptomatic patients with lower pathogen loads can be more infectious to host-seeking mosquitoes than symptomatic hosts with high pathogen loads [15, 51, 52]. This could be due to host factors that are absent in our study and related studies [16, 18, 39]. Variation in host blood quality (e.g. hematocrit) and mosquito attraction, or circulating host factors (e.g. differences in immune factors), could result in reduced infectivity of mosquitoes feeding on hosts with high pathogen burdens [15, 53]. Even the current, most frequently used ZIKV mouse models use mice lacking a large component of the innate immune system and are not likely to be representative of transmission in the field [17, 54]. Thus, our study and others should be confirmed with mosquito feeding trials on human hosts of varying viremias.

In conclusion, we demonstrate that ingesting higher doses of ZIKV increases the proportion and the rate at which mosquito populations become infectious. This, in turn, results in an increase in the relative transmission risk and the force of infection experienced by susceptible human populations. Therefore, variation in viremia, as well as the frequency distribution of hosts of different viremias, should be accounted for when estimating *R*_*0*_ and in assessing the efficacy of arbovirus prevention strategies.

## Supporting information

S1 Fig Experimental design. In each biological replicate, a total of 1,600 female *Aedes aegypti* mosquitoes were offered an infectious blood meal containing ZIKV at the final concentrations of 10^3^ PFU/mL, 10^4^ PFU/mL, 10^5^ PFU/mL or 10^6^ PFU/mL (400 females per treatment). Eighty females were offered an uninfected, control blood meal. Two hundred ZIKV-exposed engorged mosquitoes for each treatment (800 total) and 40 engorged control mosquitoes were randomly distributed into mesh-covered paper cups (40 per cup) and housed at 27°C ± 0.5°C, 70% ± 5% relative humidity, and 12:12 hr light:dark cycle for 20 days. Mosquito mortality was checked every two days. Every four days, twenty ZIKV-exposed mosquitoes per treatment group were force-salivated. After salivation, mosquito saliva, heads, and bodies were collected into separate tubes. Each tissue was tested for the presence/absence of the ZIKV using plaque assays on Vero cells. Three full biological replicates were performed.
(TIFF)

S2 Fig Days post-infection and the dissemination and transmission efficiencies. The relationship between days post-infection (4, 8, 12, 16, 20) and the proportion of infected mosquitoes with disseminated infections (A), and that are infectious (B) after exposure to four different viral doses (10^3^, 10^4^, 10^5^, and 10^6^ PFU/mL). Whiskers on each bar represent the standard error of the mean. Dose 10^4^ PFU/mL is represented by small sample sizes (< 5 infected mosquitoes at any given time point), which likely explains the decrease in the proportion of infectious mosquitoes from day 16 to 20.
(TIFF)
