# Supplementary figures and images for: Estimating the effects of variation in viremia on mosquito susceptibility, infectiousness, and R0 of Zika in Aedes aegypti
Source: PLoS Negl Trop Dis. 2018 Aug 22;12(8):e0006733. doi: 10.1371/journal.pntd.0006733 (PMC6122838; doi:10.1371/journal.pntd.0006733)

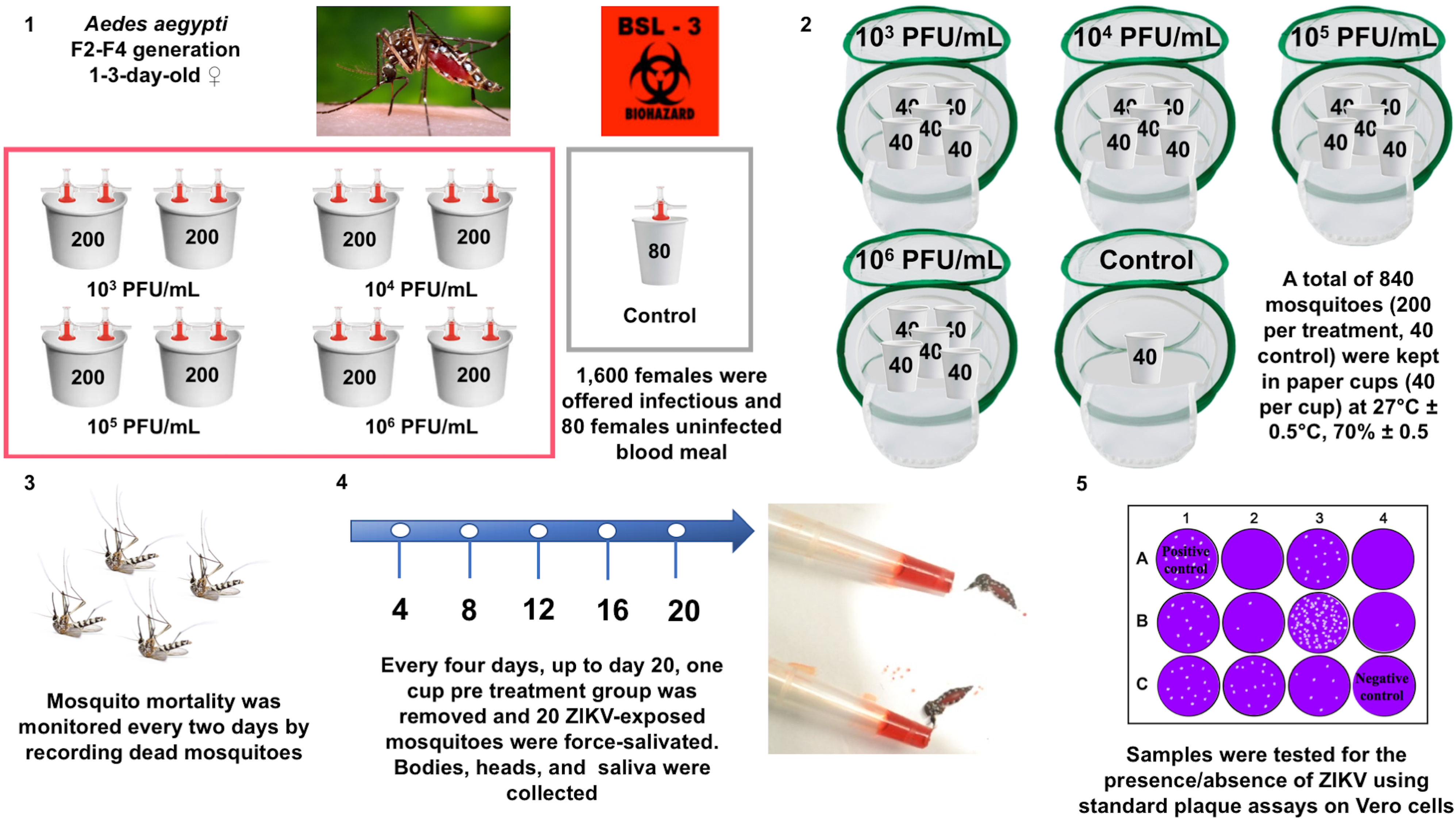

Supplement: S1 Fig — In each biological replicate, a total of 1,600 female Aedes aegypti mosquitoes were offered an infectious blood meal containing ZIKV at the final concentrations of 103 PFU/mL, 104 PFU/mL, 105 PFU/mL or 106 PFU/mL (400 females per treatment). Eighty females were offered an uninfected, control blood meal. Two hundred ZIKV-exposed engorged mosquitoes for each treatment (800 total) and 40 engorged control mosquitoes were randomly distributed into mesh-covered paper cups (40 per cup) and housed at 27°C ± 0.5°C, 70% ± 5% relative humidity, and 12:12 hr light:dark cycle for 20 days. Mosquito mortality was checked every two days. Every four days, twenty ZIKV-exposed mosquitoes per treatment group were force-salivated. After salivation, mosquito saliva, heads, and bodies were collected into separate tubes. Each tissue was tested for the presence/absence of the ZIKV using plaque assays on Vero cells. Three full biological replicates were performed. (TIFF) [file pntd.0006733.s001.tiff]

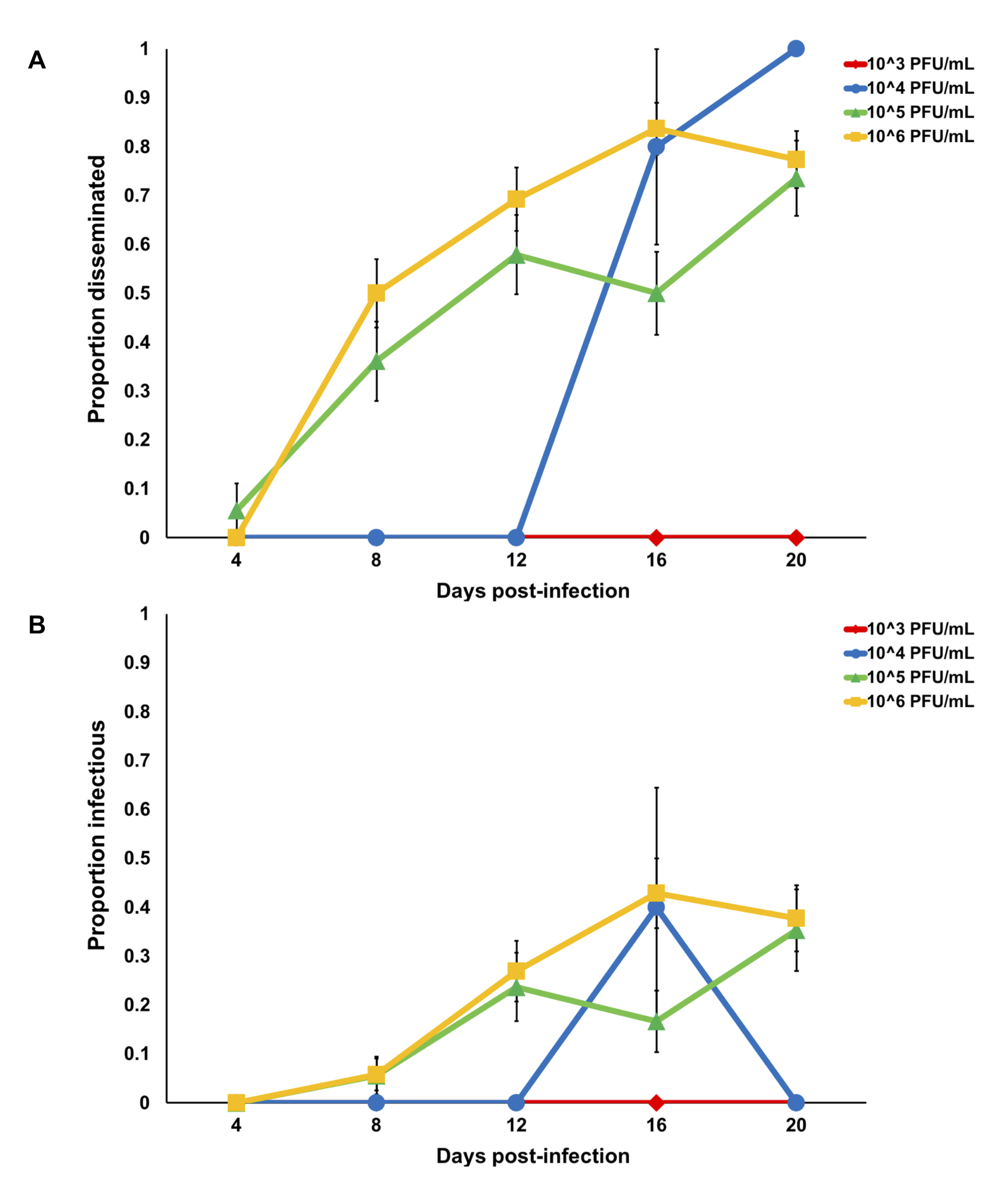

Supplement: S2 Fig — The relationship between days post-infection (4, 8, 12, 16, 20) and the proportion of infected mosquitoes with disseminated infections (A), and that are infectious (B) after exposure to four different viral doses (103, 104, 105, and 106 PFU/mL). Whiskers on each bar represent the standard error of the mean. Dose 104 PFU/mL is represented by small sample sizes (< 5 infected mosquitoes at any given time point), which likely explains the decrease in the proportion of infectious mosquitoes from day 16 to 20. (TIFF) [file pntd.0006733.s002.tiff]
